# Vicious cycle of emotion regulation and ODD symptoms among Chinese school-age children with ODD: a random intercept cross-lagged panel model

**DOI:** 10.1186/s13034-023-00579-x

**Published:** 2023-04-04

**Authors:** Wenrui Zhang, Yanbin Li, Longfeng Li, Stephen Hinshaw, Xiuyun Lin

**Affiliations:** 1 Institute of Developmental Psychology, School of Psychology, Beijing Normal University, Beijing, 100875 China; 2 Department of Psychology, College and Graduate School of Arts and Sciences, University of Virginia, Charlottesville, VA USA; 3 T. Denny Sanford School of Social and Family Dynamics, Arizona State University, Tempe, AZ USA; 4 Department of Psychology, University of California, Berkeley, CA USA; 5 Beijing Key Laboratory of Applied Experimental Psychology, School of Psychology, Beijing Normal University, Beijing, China

**Keywords:** Emotion regulation, Emotion lability, Oppositional defiant disorder, Chinese children, Random intercept cross-lagged panel model

## Abstract

A strong link between children’s emotion regulation and oppositional defiant disorder (ODD) symptoms has been documented; however, the within-person mechanisms remain unclear. Based on the self-control theory and self-regulation theory, our study investigated the longitudinal, bidirectional relationship between emotion regulation and ODD symptoms in school-age children with ODD using parent- and teacher-reported data, respectively. A total of 256 Chinese elementary school students participated in a three-wave longitudinal study spanning two years. We used the random intercept cross-lagged panel model (RI-CLPM) to investigate the concurrent and longitudinal associations between emotion regulation and ODD symptoms. Results from the RI-CLPMs revealed that ODD symptoms were negatively correlated with emotion regulation and positively correlated with emotion lability/negativity at both the between-person and within-person levels across settings. Additionally, in the school setting, emotion regulation negatively predicted subsequent ODD symptoms but not vice versa, whereas emotion lability/negativity was bidirectionally associated with ODD symptoms over time. The longitudinal associations of ODD symptoms with emotion regulation and lability/negativity were not observed in the home setting. These findings suggest a circular mechanism between children’s emotion regulation and ODD symptoms and support the view that emotion regulation, particularly emotion lability/negativity, plays an important role in the development of ODD symptoms.

## Introduction

Worldwide, one of the most common mental disorders in children under 7 years of age is oppositional defiant disorder (ODD), with a prevalence of 4.9% (95% CI [2.5, 9.5]) [1]. A recent national epidemiological survey revealed that ODD is also one of the most common mental disorders in Chinese school-age children and adolescents, with a prevalence of 3.7% (95% CI [3.5%, 3.8%]) [2]. Based on the Diagnostic and Statistical Manual of Mental Disorders-Fifth Edition (DSM-5) [3], ODD is a typical behavior disorder in early childhood characterized by a persistent angry and irritable mood, argumentative and defiant behavior, and vindictiveness. ODD generally does not subside with the passage of time [4], and it is frequently a precursor to more serious consequences [5]. Moreover, ODD is highly comorbid with other mental disorders, resulting in complex clinical presentations and generalized functional impairment in children [6, 7]. Considering the high prevalence of ODD and its long-term adverse consequences, it is essential to study the development of ODD among school-age children, as well as its precursors and consequences. Among the many factors related to ODD symptoms, it has been suggested children’s emotion regulation is critical [8, 9]. However, recent theorists have proposed that emotion regulation is a dynamic process, and the intraindividual dynamics of emotion regulation is different among individuals, while there is also a two-way interaction between emotion regulation and behavior [10]. How would children’s ODD symptoms be associated with their emotion regulation over time? Our study sought to answer this question.

### Emotion regulation and ODD symptoms

Emotion regulation is typically defined as the ability to manage and control emotional status to help individuals adapt to different external environments [11]. The ability to regulate emotions begins to develop early in life, and individual differences in emotion regulation are particularly pronounced in early and middle childhood, when ODD prevalence is high [12]. The view of the dynamic process of emotion dissonance proposes that emotion is a dynamic process, and emotion regulation is the bidirectional interplay between emotions and actions/thoughts [10]. This means that emotional regulation changes over time and has a bidirectional interplay with other factors. Therefore, a longitudinal study of the relationship between emotion regulation and ODD will be more conducive to the development of child psychopathology.

Emotion regulation is a multidimensional concept, and in the current study, we focused on emotion regulation (ER) and emotion lability (EL). Theorists have viewed ER and EL as related yet separate entities, reflecting different features of individual emotion regulation processes [13, 14]. ER is the ability to manage the experience and expression of emotions; it may include increasing or decreasing the experience of specific emotions to function well in the current environment [15]. EL is the sensitivity to emotionally triggering events and can be described as a rapid response to emotionally eliciting stimuli and the simultaneous difficulty in recovering from the emotion [16]. Children’s ER and EL have a strong negative correlation, but the two are not simply opposite ends of the same dimension [14]. Although ER and EL have been found to be significantly negatively and positively associated with ODD symptoms [18, 19], respectively, it is unclear whether this association is stable over time.

The link between emotion regulation and ODD is widely recognized, and some researchers even consider emotion dysregulation as a core feature of ODD [8, 20]. Although many studies have examined the negative correlation between emotion regulation and ODD in preschoolers, school-age children, and adolescents [19, 21–23], the longitudinal relationship between the two constructs has not been sufficiently explored. The findings of existing longitudinal studies support that children’s emotion regulation negatively predicts ODD symptoms [23]. In addition, a recent study found that children’s ODD symptoms predicted the development of emotion regulation [19]. Yet, none of these studies have examined whether there is a bidirectional relationship between emotion regulation and ODD symptoms in children. Hence, the main goal of this study is to investigate the across time and concurrent associations between emotion regulation and ODD symptoms within individuals. This research will benefit our understanding of the developmental processes of emotion regulation and ODD symptoms in children and might have implications for theoretical frameworks that aim to explain such processes.

### Emotion regulation and ODD symptoms from the perspective of self-control theory

Self-control theory [24] has long been considered crucial in the area of behavior problems in children and adolescents [25, 26], and a strong relationship between ODD and self-control deficits has been demonstrated in previous studies [27–29]. Self-control is the ability to initiate and control one’s emotions, thoughts, and behaviors in order to achieve desired outcomes or prevent undesired outcomes [30]. Self-control originates from early childhood and tends to stabilize in late childhood [31]. Good self-control allows individuals to consciously control their emotions and thus effectively regulate negative emotions [32]. On the contrary, children who have a lack of developed self-control are likely to have difficulty regulating negative emotional experiences, and consequently, have more behavioral problems and delinquency [33]. Therefore, from the perspective of self-control theory, inability to effectively control negative emotions is a primary explanation for the exacerbation of ODD symptoms. However, self-control theory can only explain how emotion dysregulation leads to the emergence of ODD, and it is difficult to explain the interaction between ODD and the development of emotion regulation. Considering these mixed findings, more broadly applicable theories are needed to explain the relationship between emotion regulation and ODD.

### Self-regulation theory: explanation of the relationship between emotion regulation and ODD

Self-regulation theory was first proposed by Bandura [34] and has attracted great attention and development in different fields. Currently, researchers believe that individual self-regulation is a dynamic process involving an individual’s ability to regulate behaviors, emotions, and thoughts related to specific goals [35–37]. This model assumes that self-regulation is an innate, biologically driven process that is influenced by experience and learning throughout the lifecycle. Any sudden state of disequilibrium, whether emotional, physical, cognitive, or interpersonal, can lead to a feeling of dissonance or discomfort that drives a desire to restore self-balance. Individuals use learned coping mechanisms to self-regulate, however, these self-regulatory strategies may be adaptive (harmless and healthy) or maladaptive (causing harm to themselves or others).

In the Test-Operate-Test-Exit (TOTE) model of self-regulation [38], self-regulation is conceptualized as three cyclic components: standards, monitoring, and operated. The standard is the expectation and direction of self-regulation, monitoring is the perception of the difference between the current state and the standard, and operated is the effort to return to the standard. The feedback loop of the three components stops when the current state satisfies the desired criterion, and if not, the loop continues. The operated component is basically equivalent to self-control, which means that self-control is included in the process of self-regulation [39]. In summary, self-regulation theory builds on self-control theory and further constructs a feedback loop. This means that while individuals control their emotions to modify their tendency to respond, they also optimize their control of emotions in turn based on the effects of the modification. Consequently, the purpose of this study was to examine whether self-regulation theory could better explain the relationship between emotion regulation and ODD symptoms.

### Situational specificity of ODD in school-age children

School-age is a crucial stage in the onset and development of ODD. Firstly, oppositional behaviors are widespread among preschoolers, and a certain amount of oppositional behavior is normal for children [41]. However, if children developed stable oppositional behavior patterns in preschool, they are likely to develop ODD in elementary school [42]. Second, the presence of ODD at school-age is likely to be a key marker for subsequent externalizing and internalizing problems [43, 44]. A large amount of evidence suggests that school-age children’s ODD behavioral symptoms (e.g., defiance, arguing) have a strong association with conduct disorder (CD) and violent behavior, while emotional symptoms (e.g., anger, sensitivity) predict depression and anxiety problems [45, 46]. Furthermore, a large, 9-year follow-up study also revealed a strong association between ODD symptoms in children at age 7 and low academic achievement at age 16 [47]. Low academic achievement, in turn, leads to poor school adjustment, involvement in delinquency and crime, and living in poverty [48]. Thus, the school-age period is a critical time for ODD transition in children, but to date, few studies have investigated the mechanisms of longitudinal changes in ODD over this period.

The school-age stage symbolizes the transfer of a child from a familiar and simple environment to a strange and complex environment, and ODD symptoms are likely to come to the fore with the drastic environment change. The family and school environments differ in their needs, activities, and purposes, which result in the situational specificity of the children’s behaviors evoked by the events [49, 50]. A laboratory study by [51] found significant differences in the behaviors exhibited by preschoolers when confronted separately by their parents and the examiner. Intriguingly, some of the children in the study exhibited disruptive behaviors only when interacting with their parents, while others exhibited disruptive behaviors only when interacting with the examiner. Thus, because of the fundamental differences in relationships and interpersonal interactions across the home and school settings [52], school-aged children may show different or even more behavioral problems in the new school environment. Based on this, it is necessary to understand the mechanisms of longitudinal changes in school-age children's ODD in both the home and school settings.

How to accurately assess mental disorders in children has always been challenging, and the most widely used method is to collect information from multiple individuals [53, 54]. Parents and teachers often are valid and reliable reporters of children’s behaviors in the home setting and school setting, respectively [55]. In previous studies, parent and teacher reported children’s behavior problems were usually combined to obtain a cross-situational index of children’s problem behaviors. The drawback of this approach, however, is that much of the information relevant to the particular situation in which the informant is located is obscured. Reference [56] followed 5-year-old kindergarten children for 8 years and compared differences in mothers’ and teachers’ assessments of children’s externalizing problem behavior. Results showed that mothers reported more severe problems regarding emotional dysregulation and oppositional defiant behavior than teachers, and this finding was replicated in other studies [55]. Given the potential differences in children’s behaviors across contexts, we examined the longitudinal associations between children’s self-regulation and ODD symptoms separately in the home and school settings, aiming to provide a more comprehensive understanding of the mechanisms of changes in children’s ODD symptoms across contexts.

### The advantages of RI-CLPMS in longitudinal studies

Given the lack of studies about the longitudinal relationship between school-age children’s ODD and emotion regulation, the goal of this study was to address this gap by employing a longitudinal design to investigate the concurrent and longitudinal, transactional associations between ODD and emotion regulation. With the advancement of statistical methods, the Cross-lagged Panel Models (CLPM) have become the standard for exploring bidirectional relationships between different constructs, especially when assessing the causal relationships between constructs [57]. However, in recent years researchers have suggested that the CLPM is flawed by compounding between-person and within-person effects, which are not only conceptually different but also different in magnitude and direction [58, 59]. The RI-CLPM proposed by Hamaker (2015) is one of the effective methods to solve this defect. The model accounts for compounding due to a trait-like stability by incorporated random intercepts and also captures the overall stability of the relationship between constructs at the between-person level by allowing for intercepts to covary. Accordingly, we used RI-CLPM for analysis, which allowed us to better understand the relationship between ODD and emotion regulation at the between-person and within-person levels.

## The present study

The present study aimed to contribute to our understanding of the association between emotion regulation and ODD symptoms in four important ways. First, although self-control theory has made an important contribution to explaining the relationship between children’s emotion regulation and behavioral problems, we believe that self-regulation theory can better explain the relationship between children’s emotion regulation and ODD symptoms. Therefore, we hypothesized that child emotion regulation not only predicts ODD symptoms longitudinally, but that there is a reciprocal predictive relationship between the two. Second, emotion regulation is a multidimensional concept, so we tested the ER and EL in relation to ODD symptoms and expected to find different associations. Third, based on evidence of differences in children's performance across situations, we also analyzed differences in the relationship between children's emotion regulation and ODD symptoms under parent and teacher reports. Finally, based on recent advances in longitudinal association analysis, the present study used RI-CLPMs for analysis, which allowed for the separation of within-person and between-person processes in the association between emotion regulation and ODD.

## Method

### Participants

Participants were recruited from 8 elementary schools in the north (Beijing), 2 elementary schools in the east (Shandong), and 4 primary schools in the southwest (Yunnan) of Mainland China. Assessments were conducted annually over a two-year period (three waves, Time 1–Time 3) in 2013–2014. One child per family participated in the study. At baseline (Time 1), participants included 256 families from Grade 1 to Grade 6 (range 6–13 years, 186 boys; *M*_age_ = 9.56 years, SD = 1.58), 245 families participated at Time 2 (181 boys; *M*_age_ = 10.26 years, SD = 1.28), and 208 families participated at Time 3 (126 boys; *M*_age_ = 11.46 years, SD = 1.32). The participant retention rates were 95.7% and 81.3% at the Time 2 and Time 3 assessments, respectively. Attrition was mainly due to students transferring to other schools. In the remaining families, the majority of mothers (77.0%) and fathers (79.5%) had high school diplomas or above. All the children and parents in the study were Han Chinese.

In this study, to ensure that missing data did not bias the results, Little’s Missing Completely at Random test based on all variables was computed, χ^2^ (519) = 468.095, *p* = 0.947, indicating that the sample attrition across the study’s duration was completely at random [60]. Second, for model estimations, we applied Full Information Maximum Likelihood (FIML), which estimates model parameters using all available information, producing unbiased estimates for non-normality for indicator variables when data are not missing completely at random (Little and Rubin, 2002).

### Procedure

In April 2013 (T1), invitation letters and consent forms were sent to the class head teachers of Grades 1 to 6 classrooms through school psychologists. A total of 187 teachers signed informed consent and agreed to participate in the study. Based on descriptive criteria in the DSM-IV-R [61], teachers were asked to nominate any of their students who might have symptoms of ODD. Only those children rated as showing four or more symptoms were considered candidates for further investigation. Then, two clinical psychologists from [BLINDED] University interviewed class head teachers to individually confirm each candidate child’s ODD diagnosis, using a semi-structured interview. Inclusion criteria were the following: (a) the child exhibited four or more symptoms of ODD described in DSM-IV-TR; (b) the child’s identified symptoms had lasted for six months or more; and (c) the child demonstrated significant impairment across psychosocial functional domains [62]. By this method, children with only ODD, as well as children with ODD comorbid CD or attention deficit/hyperactivity disorder (ADHD), were included in the study. Ultimately, 305 of 7966 (3.8%) children from 14 elementary schools were identified as exhibiting ODD and were invited to participate in the study. After invitations and informed consent letters were sent to the children's parents, 259 families agreed to participate in the follow-up study. Excluding other invalid questionnaires (i.e., missing data on more than half of the items in the questionnaire), the final ODD sample consisted of 256 children. Data at Time 2 (T2) and Time 3 (T3) were collected approximately 1 and 2 years later, respectively.

Each participating child was asked to forward a package containing a parental survey to his or her primary caregiver and to return their completed survey to the class head teacher within a week. The homeroom teacher completed a questionnaire in their office to assess each child’s ODD symptoms. Each participant, including parents, children, and teachers, received a small payment (equivalent to $8) as compensation for their participation. Furthermore, each participating family was provided with the opportunity for consultation or treatment from psychiatrists in Anding Hospital, or from psychological counselors and family therapists at the Center of Family Study and Therapy. The Institutional Review Board of [BLINDED] University approved the protocol of the present study, including the consent procedure.

### Measures

#### Teacher-rated ODD symptoms

Children’s ODD symptoms in the school context were reported by teachers working with clinical psychologists, using the eight items from the diagnostic criteria listed in DSM-IV-R [61]. The Chinese version of these items have been validated by [63], showing good test–retest reliability and high internal consistency in Chinese children and adolescents (e.g., “often deliberately annoys people”). Each teacher rated the children’s ODD symptoms with a dichotomous scale (0 = no, 1 = yes). The summed scores of eight items were used as the indicator of ODD symptoms in school. The higher the total score, the more ODD symptoms the child showed. Because ODD symptoms reported by teachers at Time 1 were used to help diagnose ODD, the relevant scores ranged between 4 and 8 instead of 0–8, so the KR-20 Coefficient was calculated for teacher rated ODD symptoms [64]. In this study, the KR-20 Coefficients were 0.93, 0.90, 0.91 for T1 to T 3, respectively.

#### Parent-rated ODD symptoms

Children’s ODD symptoms were also reported by their parents (father or mother) using the eight items from the diagnostic criteria listed in DSM-IV-R [61]. The Cronbach’s coefficients *α* were 0.85, 0.83, 0.85 for T1 to T3, respectively.

#### Teacher-rated child emotion regulation

Teachers reported children’s emotion regulation using the Emotion Regulation Checklist [65]. The Chinese version of the ERC has shown good reliability and validity in Chinese elementary school students [66]. The ERC contains 23 items indicating how frequently a child manifests affective behavior that is rated on a 4-point scale (1 = *never*, 4 = *always*). The checklist is divided into two subscales: Emotion Regulation and Lability/Negativity. The Emotion Regulation subscale, consisting of 8 items, assesses the social appropriateness of child’s emotions, including emotion understanding and empathy, and includes items such as “Can modulate excitement in emotionally arousing situations”. In this study, the Cronbach’s coefficients *α* were 0.71, 0.71, 0.67 for T1 to T3, respectively. The Emotion Lability/Negativity subscale, consisting of 15 items, measures mood swings, anger outbursts, and intensity of both positive and negative emotions. The Lability/Negativity scale includes items such as “Exhibits wide mood swings”. In this study, the Cronbach’s coefficients *α* were 0.81, 0.81, 0.82 for T1 to T3, respectively.

#### Parent-rated child emotion regulation

Parents also used ERC to assess the child’s emotion regulation. The Cronbach’s coefficient *α* of Emotion Regulation subscale T1 to T3 were 0.68, 0.69 and 0.73, respectively. The Cronbach’s coefficient *α* of Emotion Lability/Negativity subscale T1 to T3 were 0.75, 0.77 and 0.78, respectively.

#### Demographics

Parents provided information about child gender, child age, and family monthly income and these variables were included as covariates. Family monthly income was coded as 1 (≤ 2000 Chinese yuan, 7.8%), 2 (2000–5000 Chinese yuan, 34.0%), 3 (5000–10,000 Chinese yuan, 29.8%), 4 (10,001–30,000 Chinese yuan, 19.5%), and 5 (≥ 30,001 Chinese yuan, 5.1%), and 4.7% did not answer the question.

#### Data analysis strategy

First, descriptive analyses and correlations were calculated using SPSS 24.0 [67]. Then, the Intraclass Correlation Coefficients (ICCs) for all study variables were computed. A high ICC (> 0.50) indicates proportionately higher between-person variance, while a low ICC (< 0.50) indicates proportionately higher within-person variance [68].

Second, confirmatory factor analysis (CFA) was used to investigate the longitudinal measurement invariance for study variables. The respective items of study variables were measured as the observed indicators. Among them, the Emotion Lability/Negativity subscale contains 15 items. To control for inflated measurement errors due to multiple items, the Emotion Lability/Negativity subscale were represented by three parcels created by the item-construct balance approach [69].

Third, the RI-CLPM was used to examine between-person and within-person associations between variables. The RI-CLPM incorporated the random intercept into the equation based on CLPM, which can explain the confounding due to trait-like stability [58]. In the RI-CLPM, the random intercepts responded to stable individual differences, while the coefficients of the autoregression and lag effects represent within-person patterns of change. Four RI-CLPM were constructed in this study: Model 1 examined the relationship between teacher reported ODD symptoms and ER; Model 2 examined the relationship between teacher reported ODD symptoms and EL; Model 3 examined the relationship between parents reported ODD symptoms and ER; Model 4 examined the relationship between parents reported ODD symptoms and EL. Children’ sex, age, and their family’s socioeconomic status were entered in all RI-CLPMs as time-invariant covariates and were regressed on all variables at all three time points. To determine the most parsimonious model, two types of models were tested respectively, including a fully constrained model with all cross-lagged paths and autoregressive paths set to be time-invariant as the baseline model and a fully unconstrained model.

This study used FIML estimation of all CFA and RI-CLPM models in Mplus 8.3 [70]. For each model, chi-square values, the comparative fit index (CFI), standardized root mean square residual (SRMR), and root-mean-square error of approximation (RMSEA) were reported. Model fit is considered acceptable if CFI is greater than 0.90 and RMSEA and SRMR are less than 0.08 [71]. For reasons of parsimony, when the model fit of the constrained model was not significantly worse than that of the unconstrained model, the constrained (i.e., more parsimonious) model was retained. The CFI and RMSEA were used as two criteria for model comparisons. When △CFI < 0.010 and △RMSEA < 0.015 between the constrained and unconstrained models, the null hypothesis of invariance was accepted and the constrained model was chosen [72].

### Power analysis

Considering the small sample size of this study, it is necessary to conduct power analyses according to the suggestions of previous studies [73]. To examine our ability to identify minimum observed effects (*α* = 0.05) across parameters under the current sample, a post hoc Monte Carlo simulation (10,000 simulated samples) was run. The results indicated that power for a small effect (β, r ≥ 0.10) was sufficient (≥ 0.80) for all constrained RI-CLPM models, but very small effects (β, r < 0.10) could not be detected. In addition, bias in parameter estimates and standard errors was small (< 0.10), which also indicates that the sample size is sufficient [74].

## Results

### Descriptive statistics, correlation, and intraclass correlation

Table 1 contains the results of descriptive statistics for all variables, and correlations between the two variables within and across measurement occasions. The effect sizes of the correlation coefficients were based on the absolute values, wherein *r* > 0.10 is small, *r* > 0.30 is moderate, and *r* > 0.50 is large [75]. The results of the correlation analysis showed that at the same time point, teacher-reported ODD symptoms were negatively associated with ER in a small to moderate effect (–0.28 ~ –0.44), while teacher reported ODD symptoms were positively associated with EL in a moderate to large effect (0.49 ~ 0.77). Parent reported ODD symptoms were negatively associated with ER in a moderate effect (–0.33 ~ –0.50), while parent reported ODD symptoms were positively associated with EL in a large effect (0.58 ~ 0.68). Furthermore, the ICCs for teacher-reported ODD symptoms, ER, and EL were 0.46, 0.40 and 0.44, respectively, which were 0.48, 0.35 and 0.34 for parental reports, indicating that there were sufficient within-person variances to use RI-CLPMs to investigate within-person changes over time.

**Table 1:** Descriptive statistics and correlation matrix of variables over three time points

Variable	M	SD	1	2	3	4	5	6	7	8	9	10	11	12	13	14	15	16	17	18	
1. T1 ODD symptoms (T)	5.48	1.38	–																		
2. T2 ODD symptoms (T)	3.47	2.96	.41***	–																	
3. T3 ODD symptoms (T)	3.16	3.04	.39***	.66***	–																
4. T1 Emotion Regulation (T)	15.54	3.85	−.28***	−.32***	−.21**	–															
5. T2 Emotion Regulation (T)	15.24	3.93	−.23***	−.43***	−.31***	.45***	–														
6. T3 Emotion Regulation (T)	14.81	3.72	−.27***	−.40***	−.44***	.30***	.48***	–													
7. T1 Emotion Lability/Negativity (T)	40.88	8.26	.49***	.55***	.46***	−.47***	−.34***	−.37***	–												
8. T2 Emotion Lability/Negativity (T)	37.11	9.64	.36***	.75***	.56***	−.34***	−.51***	−.37***	.57***	–											
9. T3 Emotion Lability/Negativity (T)	36.20	9.21	.30***	.55***	.77***	−.28***	−.26**	−.54***	.49***	.51***	–										
10. T1 ODD symptoms (P)	2.65	2.53	.21***	.45***	.37***	−.28***	−.32***	−.33***	.34***	.36***	.32***	–									
11. T2 ODD symptoms (P)	1.99	2.28	.16*	.37***	.28***	−.09	−.22**	−.27***	.26***	.31***	.23**	.56***	–								
12. T3 ODD symptoms (P)	2.13	2.39	.14	.21**	.31***	−.19**	−.23**	−.23**	.24**	.16*	.24**	.48***	.38***	–							
13. T1 Emotion Regulation (P)	26.32	3.44	−.06	−.16*	−.21*	.23***	.27***	.25**	−.19**	−.10	−.06	−.50***	−.28***	−.34***	–						
14. T2 Emotion Regulation (P)	26.48	3.59	−.06	−.25***	−.20*	.13	.33***	.28***	−.20**	−.27***	−.05	−.36***	−.45***	−.26***	.43***	–					
15. T3 Emotion Regulation (P)	26.17	3.31	−.04	−.02	−.05	.12	.18*	.18*	−.03	−.02	.04	−.17*	−.12	−.33***	.37***	.29***	–				
16. T1 Emotion Lability/Negativity (P)	34.20	6.39	.13	.32***	.22**	−.24***	−.24**	−.29***	.28***	.28***	.20*	.68***	.43***	.42***	−.47***	−.35***	−.26**	–			
17. T2 Emotion Lability/Negativity (P)	33.27	6.59	.10	.32***	.28***	−.16*	−.25***	−.22**	.30***	.39***	.21**	.48***	.58***	.31***	−.30***	−.46***	−.14	.47***	–		
18. T3 Emotion Lability/Negativity (P)	32.40	6.71	.10	.19*	.28***	−.18*	−.20**	−.20*	.24**	.24**	.25**	.35***	.34***	.66***	−.22**	−.30***	−.47***	.41***	.32***	–	

M = mean; SD = standard deviation. T = Teache, P = Parents. *p < .05; **p < .01; ***p < .001

#### Longitudinal measurement invariance

As shown in Table 2, the configural, metric, and scalar invariance models were tested sequentially for the ODD symptom and ERC. The results show that the configural, metric, and scalar invariance models are not significantly different in all variables. The achievement of scalar invariance for all study variables supported meaningful comparisons across the three measurement waves.

**Table 2:** Model fit and comparison for measurement invariance

Variable	Models	*χ*^2^ (*df*)	CFI	SRMR	RMSEA	△CFI	△RMSEA
**Teacher ratings**							
*ODD symptoms*							
	Configural invariance	3.997 (48)	0.977	0.064	0.066	–	–
	Metric invariance	4.814 (62)	0.972	0.068	0.075	−0.005	0.009
	Scalar invariance	5.433 (78)	0.964	0.068	0.080	−0.008	0.005
Emotion regulation							
	Configural invariance	1.567 (51)	0.971	0.045	0.029	–	–
	Metric invariance	1.443 (65)	0.971	0.050	0.026	0.000	−0.003
	Scalar invariance	1.466 (81)	0.962	0.051	0.026	−0.009	0.000
Emotion lability/negativity							
	Configural invariance	0.000 (0)	1.000	0.006	0.033	–	–
	Metric invariance	0.673 (4)	1.000	0.006	0.033	0.000	0.000
	Scalar invariance	2.043 (10)	0.995	0.006	0.034	−0.005	0.001
**Parents ratings**							
*ODD symptoms*							
	Configural invariance	165.114 (48)	0.939	0.038	0.053	–	–
	Metric invariance	193.858 (62)	0.931	0.039	0.051	−0.008	−0.002
	Scalar invariance	222.833 (78)	0.923	0.040	0.048	−0.008	−0.003
*Emotion regulation*							
	Configural invariance	73.572 (51)	0.964	0.043	0.052	–	–
	Metric invariance	87.333 (65)	0.962	0.054	0.045	−0.002	−0.007
	Scalar invariance	89.273 (81)	0.962	0.062	0.044	0.000	−0.001
*Emotion lability/negativity*							
	Configural invariance	0.000 (0)	1.000	0.030	0.028	–	–
	Metric invariance	8.491 (4)	0.992	0.030	0.020	−0.008	−0.008
	Scalar invariance	13.177 (10)	0.995	0.030	0.013	0.003	−0.007

CFI = Comparative Fit Index; RMSEA = Root Mean Square Error of Approximation; SRMR = Standardized Root Mean Square Residual

#### RI-CLPM between emotion regulation and ODD symptoms

As shown in Table 3, model fit of the fully constrained and fully unconstrained models for Models 1–4 was acceptable, with no significant differences between fully constrained and fully unconstrained models (i.e., △CFI < 0.010 and △RMSEA < 0.015). Based on the principle of model parsimony, the fully constrained models for Models 1–4 were selected as the final RI-CLPMs.

**Table 3:** Model fit of RI-CLPMs

Models		*χ*^2^ (df)	CFI	SRMR	RMSEA	△CFI	△RMSEA
Taecher Ratings of ODD Symptoms and Emotion Regulation	Model 1	19.096 (17)	0.994	0.040	0.023 [0.000, 0.065]	–	–
	RI-CLPM						
	fixed						
	Model 1	9.048 (13)	1.000	0.026	0.013 [0.000, 0.044]	0.006	−0.010
	RI-CLPM						
	free						
Taecher Ratings of ODD Symptoms and Emotion Lability/Negativity	Model 2	25.637 (17)	0.986	0.062	0.046 [0.000, 0.080]	–	–
	RI-CLPM						
	fixed						
	Model 2	14.207 (13)	0.988	0.049	0.034 [0.000, 0.069]	0.002	−0.012
	RI-CLPM						
	free						
Parents Ratings of ODD Symptoms and Emotion Regulation	Model 3	33.466 (17)	0.994	0.039	0.034 [0.000, 0.075]	–	–
	RI-CLPM						
	fixed						
	Model 3	0.634 (13)	1.000	0.012	0.029 [0.000, 0.058]	0.006	−0.005
	RI-CLPM						
	free						
Parents Ratings of ODD Symptoms and Emotion Lability/Negativity	Model 4	29.140 (17)	0.996	0.058	0.045 [0.015, 0.080]	–	–
	RI-CLPM						
	fixed						
	Model 4	0.118 (13)	1.000	0.024	0.034 [0.000, 0.072]	0.004	−0.011
	RI-CLPM						
	free						

RI-CLPM free = Fully Unconstrained Random Intercept Cross-Lagged Panel Model; RI-CLPM fixed = Random Intercept Cross-Lagged Panel Models with Time Invariance Constrains on the Autoregressive Stabilities and the Cross Lagged Effects; CFI = Comparative Fit Index; RMSEA = Root Mean Square Error of Approximation; SRMR = Standardized Root Mean Square Residual

**Fig. 1:**
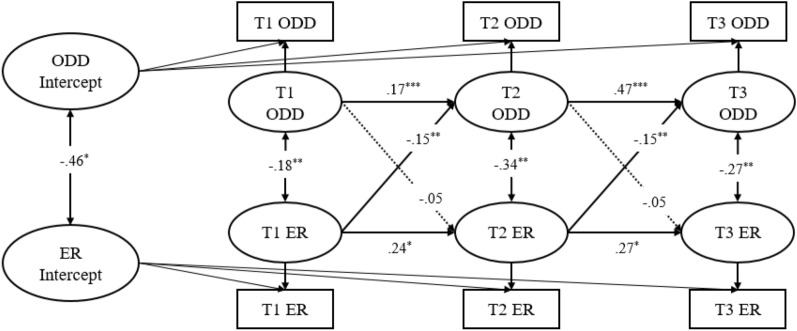
RI-CLPM for teacher reported ODD Symptoms (ODD) and Emotion Regulation (ER) across three measurement occasions (Model 1). Standardized estimates, significant (solid lines) and not significant (dotted lines) paths are included. For clarity, the effects of the covariates child gender, child age, and family monthly income are not shown. *p < .05; **p < .01; ***p < .001

Figure 1 demonstrates the RI-CLPMs between teacher reported ODD symptoms and ER (Model 1). At the between-person level, the association/correlation of ODD symptoms and ER was negative (*r* = –0.46, *p* < 0.05), and moderate in effect size. This means that individuals with low ER generally had higher levels of ODD symptoms across the three time points. At the within-person level, ODD symptoms and ER similarly showed a significant negative correlation at each of the three time points (*r* = –0.18 ~ -0.34, *p* < 0.01). This indicated that the possibility of reverse deviation of ODD symptoms increases when child’s ER levels deviated from the individual norm at the same time point. Meanwhile, there were significant and positive autoregressive effects for both constructs (*p* < 0.05). This means that when a child’s ODD symptom score was below the expected level, the subsequent ODD symptom score may again be below their expected level [58]. Furthermore, significant and negative cross-lagged effects were observed from ER to subsequent ODD symptoms (*r* = –0.15, *p* < 0.01), but not from ODD symptoms to later ER (*p* > 0.05). This suggests that the within-person deviation for ER at each age has a negative predictive effect on the within-person deviation for ODD symptoms at subsequent time points.

**Fig. 2:**
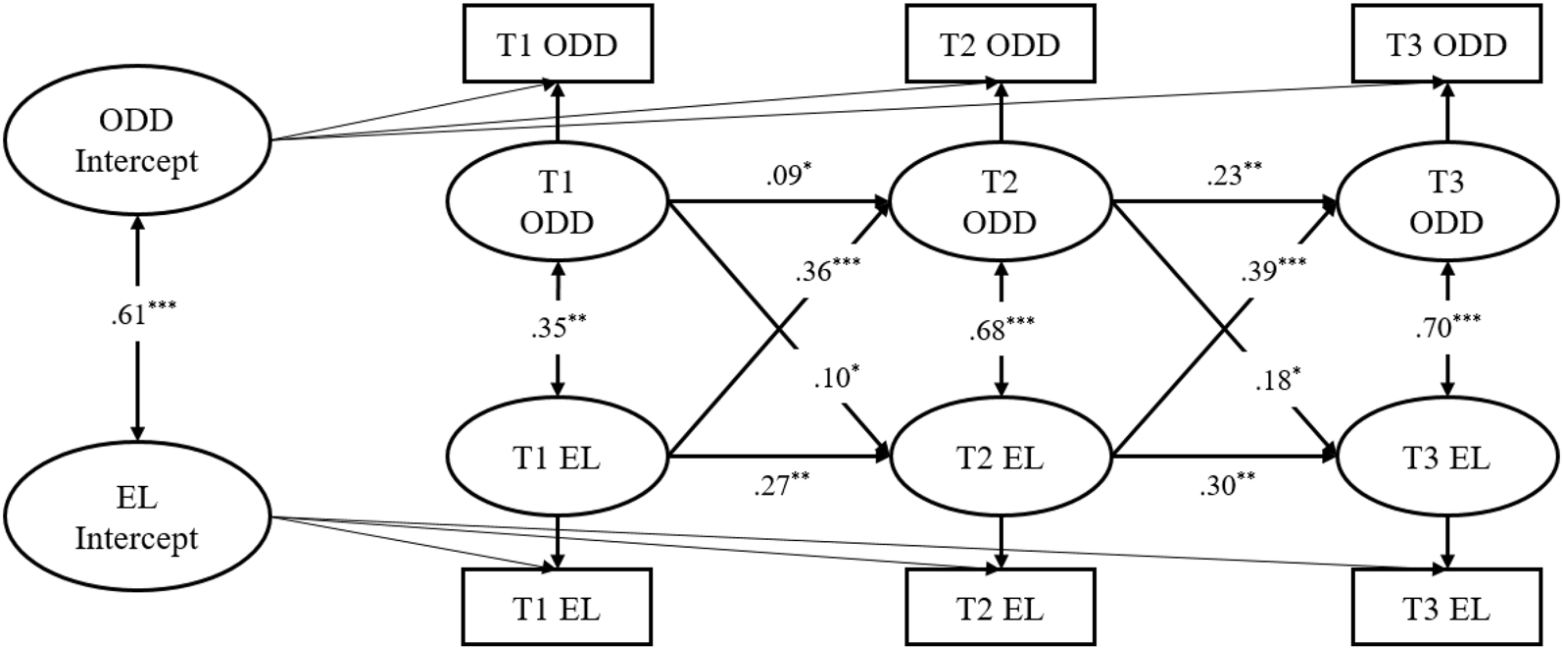
RI-CLPM for teacher reported ODD Symptoms (ODD) and Emotion Lability/Negativity (EL) across three measurement occasions (Model 2). Standardized estimates and significant (solid lines) paths are included. For clarity, the effects of the covariates child gender, child age, and family monthly income are not shown. *p < .05; **p < .01; ***p < .001

Figure 2 demonstrated the RI-CLPMs between teacher reported ODD symptoms and EL (Model 2). At the between-person level, the correlation between ODD symptoms and EL was positive (*r* = 0.61, *p* < 0.001), and large in effect size. This means that individuals with high EL generally had higher levels of ODD symptoms across the three time points. At the within-person level, ODD symptoms and EL similarly showed a significant positive correlation at each of the three time points (*r* = 0.35 ~ 0.70, *p* < 0.01). This indicated that the possibility of the same deviation of ODD symptoms increases when a child’s EL levels deviated from the individual norm at the same time point. Meanwhile, there were significant and positive autoregressive effects for both constructs (*p* < 0.05). Furthermore, significant and positive cross-lagged effects from EL to subsequent ODD symptoms were observed across the time points (*r* = 0.36–0.39, *p* < 0.001), while significant and positive cross-lagged effects from ODD symptoms to subsequent EL were also observed across the time points (*r* = 0.10–0.18, *p* < 0.05). This suggested that the within-person deviation for EL at each time has a positive predictive effect on the within-person deviation for ODD symptoms at subsequent time points, and vice versa.

**Fig. 3:**
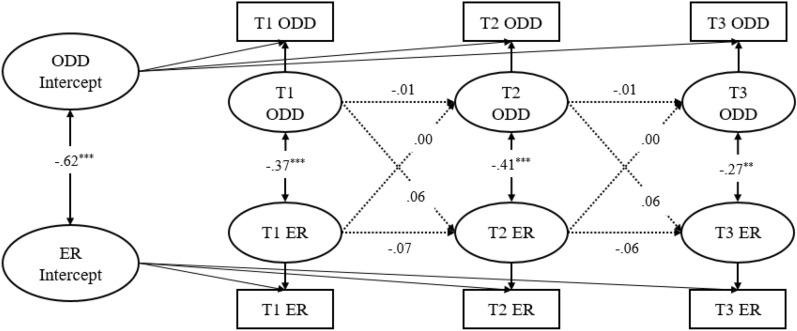
RI-CLPM for parent reported of ODD Symptoms (ODD) and Emotion Regulation (ER) across three measurement occasions (Model 3). Standardized estimates, significant (solid lines) and not significant (dotted lines) paths are included. For clarity, the effects of the covariates child gender, child age, and family monthly income are not shown. *p < .05; **p < .01; ***p < .001

**Fig. 4:**
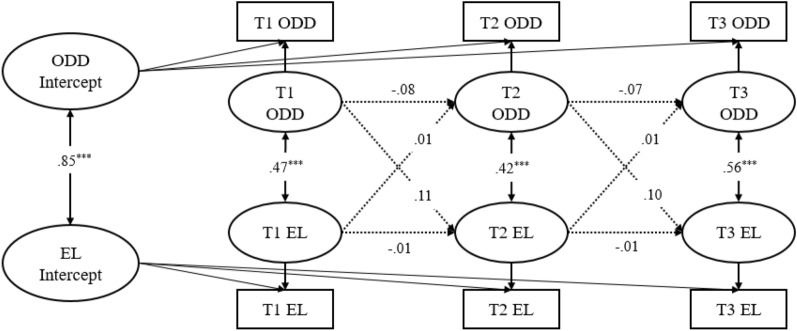
RI-CLPM for parent reported of ODD Symptoms (ODD) and Emotion Lability/Negativity (EL) across three measurement occasions (Model 4). Standardized estimates, significant (solid lines) and not significant (dotted lines) paths are included. For clarity, the effects of the covariates child gender, child age, and family monthly income are not shown. *p < .05; **p < .01; ***p < .001

Figure 3 demonstrated the RI-CLPMs between parent reported ODD symptoms and ER (Model 3), and Fig. 4 demonstrated the RI-CLPMs between parents reported ODD symptoms and EL (Model 4). At the between-person level, the correlation between ODD symptoms and ER was negative (*r* = –0.61, *p* < 0.001), whereas the correlation between ODD symptoms and EL was positive (*r* = 0.85, *p* < 0.001). At the within-person level, ODD symptoms and ER similarly showed a significant negative correlation within each of the three time points (*r* = –0.27 ~ –0.41, *p* < 0.01), whereas ODD symptoms and EL similarly showed a significant positive correlation within each of the three time points (*r* = 0.42 ~ 0.56, *p* < 0.001). However, the autoregressive and cross-lagged effects of ODD symptoms and ER as well as ODD symptoms and EL were not significant (*p* > 0.05). This indicated that at the within-person level, parent reported ODD symptoms, ER, and EL at different time points were not influenced by each other.

## Discussion

The present study used three waves of longitudinal data and RI-CLPM to test the associations between emotion regulation and ODD symptoms in Chinese children with ODD. Three main findings are discussed. First, we tested whether emotion regulation was bidirectionally related to ODD symptoms over time. We found a vicious cycle between teacher reported EL and ODD symptoms, which provides support for self-regulation theory to better explain the longitudinal development of ODD symptoms compared to self-control theory. Second, we were surprised to find that this vicious circle was present only in the relationship between teacher-reported EL and ODD symptoms. This supports the view that emotion regulation, particularly EL, is a core feature of ODD [8, 76]. Third, interestingly, we also found significant differences between teacher and parent reports, with the lack of a longitudinal association between parent reported emotion regulation and ODD symptoms. This reveals the situational specificity of school-aged children with ODD and emphasizes the need to collect information from multiple sources about children.

## The vicious cycle between emotion regulation and ODD symptoms

Our results are consistent with previous studies on between-person levels of emotion regulation and ODD symptoms (Chen et al. 2021; Lin et al. 2019; Mitchison et al. 2020; Yu et al. 2021). In the three-wave RI-CLPMs, we observed that the stable trait-like component of ER was moderately and negatively associated with ODD symptoms, whereas the stable trait-like components of EL were largely and positively associated with ODD symptoms. This finding implies that children with low ER and high EL tend to have more ODD symptoms during the school-age years, and vice versa. The moderate to large concurrent relationship between these variables are consistent with previous studies [19, 21, 22, 23].

Our results are similar to previous longitudinal studies at the between-person level [19, 21, 23], where within-person level teacher-reported emotion regulation predicted subsequent ODD symptoms, which supports the idea of self-control theory. Self-control problems are a core component of many mental disorders, and patients frequently exhibit a pattern of choosing immediate gratification regardless of long-term adverse effects [77]. Emotional regulation is a form of self-control [78], and children with difficulty in emotion regulation also show such a pattern. That is, when ODD children experience negative emotions (e.g., anger), they immediately express their emotions and act accordingly, without attempting to control them. There was evidence that children who received the self-control training intervention had significantly fewer teacher-reported symptoms of ODD compared to the control group [27]. This shows the importance of good emotion regulation in the optimal development of early social cognitive process patterns in children and as a protective factor against psychological disorders in children.

Additionally, our results showed that at the within-person level ODD symptoms predicted subsequent EL in the school setting, which supports the self-regulation theory. Specifically, a within-person increase in ODD symptoms predicted a within-person increase in EL at a later time point. This cross-lagged pathway supports the feedback mechanism of the TOTE model of self-regulation: children’s emotion regulation difficulties predict subsequent ODD symptoms, which in turn predict more emotion regulation difficulties. However, the absence of bidirectional effects in previous studies of CLPM [19], suggests that the vicious cycle of EL and ODD symptoms may exist only at the within-person level. Although self-regulation theory is unable to make explicit assumptions about the temporal continuum of high and low emotion regulation, recent research has found that the ability to self-regulate relies on a limited source of energy, namely self-regulation [79, 80]. This mean is that self-regulation requires effort but there is a limited capacity of effort that we can exert to regulate ourselves. Self-regulation is depleted when individuals engage in self-regulatory activities, such as changing mood and behavior. Thus, children's depletion of self-regulatory resources in emotion regulation leads to lower performance in subsequent self-regulation to suppress ODD symptoms, and children's depletion of self-regulatory resources in suppressing ODD symptoms leads to lower performance in emotion regulation, a phenomenon known as self-exhaustion [81]. Therefore, we presume that EL and ODD symptoms form a vicious circle. Children with ODD who have deficits in emotional self-regulation may be more likely to use maladaptive coping mechanisms (such as sudden tantrums) when experiencing dysregulation. Instead of alleviating the dysregulation, maladaptive coping mechanisms further impair emotion regulation in children with ODD [40].

### The more important role of emotion lability/negativity in the development of ODD symptoms

The current study supports previous research at the intra-individual level that there is a longitudinal association between emotion regulation and the development of ODD symptoms in children [23], and that the lack of effective emotion regulation is a critical factor in the exacerbation of ODD symptoms. More importantly, our results suggest that EL seems to have a more profound effect on ODD development compared to the emotion regulation dimension. On the one hand, only the EL had a vicious circular relationship with ODD symptoms; on the other hand, the effect size of the emotion regulation dimension with ODD symptoms was also significantly smaller than that of the EL. Similar results were found in a recent study, where children rated above the cut-off for high EL seemed to have more severe ODD symptoms [55]. It is well established that ER and EL, although intrinsically related, can be distinguished from each other because EL is the process that triggers emotions, whereas ER is the process that controls emotions [17]. The core feature of ODD might be that children trigger emotions more frequently and that the frequency increases as symptoms get worse. In addition, children’s ability to control existing emotions would not result in ODD symptoms and may even gradually return to the level of normal children. Controlling existing emotions can also be observed in some children characterized by persistent negative emotions (e.g., anger), nevertheless, more of a callous-unemotional trait accompanied by more aggressive, vindictive behavior [82, 83].

The results of the study also suggest that measuring levels of emotion regulation across different dimensions can help better identify childhood mental disorders. Emotion regulation is a transdiagnostic construct that underpins many forms of psychopathological categories [84]. Although emotion regulation problems are common in many childhood mental disorders, the emotion lability dimension is highly specific to ODD symptoms [21]. [85] also found that among children with ADHD, those with comorbid ODD scored significantly higher on the emotional lability dimension compared to those without.

### Differences between teacher and parent reports

An interesting finding of this study was that significant differences emerged in the longitudinal relationship between teacher and parent reported children’s emotion regulation and ODD symptoms. At the within-person level, there were neither cross-lagged effects nor autoregressive effects for parent reported child emotion regulation and ODD symptoms. This particular finding might be explained that in the Eastern cultural context, parents prefer to weaken the reaction by downplaying an upsetting event and supporting the child to accept the situation [86]. Thus, in a tightly controlled home environment with parents, children learn to suppress the expression of negative emotions and ODD symptoms. However, when children are in a school environment that lacks parental supervision and discipline, their emotional dysregulation and ODD symptoms arise and are observed by teachers [87] found that in collectivist countries, children tend to hide emotions in front of family members due to pro-social and self-protective motives. However, when interacting with peers, these children are much less likely to hide emotions. Therefore, the present study suggested that school teachers may be more appropriate informants than parents for ODD symptoms in school-aged children. First, teachers are frequently the primary informants of children’s behavior at school because they are able to observe children extensively at various times and situations [88]. Therefore, the symptoms of ODD in school-age children are highly visible and easily identifiable by teachers. Parents, by contrast, may not recognize ODD symptoms as a mental disorder because they spend so much time with their children. Second, the identity characteristics of parents and teachers lead to different interpretations of the same behavior of the child, thus affecting the accuracy of the reports. For example, overly controlling and controlling parents may perceive their child’s refusal behavior as a provocation of their authority. Especially for parents who also have psychological problems, negative perceptions greatly influence their perceptions of their children's behavior [89]. Lastly, for children with ODD, the beginning of their symptoms mostly coincides with school entry, leading to an emotional dysregulation that may be particularly disruptive in the classroom setting [21]. Therefore, for children at this stage, ODD symptoms are quite evident in schools. In addition, [90] has also found that teachers had higher reliability in assessing Malaysian elementary school children with ODD in symptoms compared to parents, and there was also an advantage in the validity of all model factor models.

### Limitations

The current work is novel in that it uses a longitudinal design to investigate the symptoms of emotional regulation and ODD in children separately in both home and school contexts, with disentangle between between-person and within-person processes during analysis. However, some limitations should be noted. First, there was a clear distinction between teachers’ and parents’ rated ODD symptoms, although these children were diagnosed which ODD by psychologists. This may be related to the social desirability that parents tend to report high emotion regulation and low ODD symptoms in their children. Future work should employ more observation-based measures, particularly for ODD symptoms and emotion regulation across contexts. Second, the duration of follow-up of children in this study was only two years and the number of follow-ups was only three time. Considering that school-age is a sensitive time for the rapid development of self-regulation in children, changes may occur within 6 months [91]. Future work should use more intensive assessments to examine the patterns of changes in children’s emotion regulation and ODD symptoms throughout childhood and beyond over longer periods of time. Third, this study was conducted on a single time scale. Dynamic systems theory suggests that development can be observed on different time scales [92], such as daily, monthly, or yearly development. Therefore, whether there is still an interaction between emotion regulation and ODD symptoms at different time scales needs to be explored. Fourth, the sample size of this study is relatively small, and the generalizability of the conclusions obtained needs to be further verified by future studies. Fourth, although the power of the sample in this study is sufficient for testing small effects, larger samples are needed for verification of smaller effects. Fifth, since there are angry-related items in both Emotion Regulation Checklist and diagnostic criteria for ODD, this might lead to the possibility that the correlation between ODD symptoms and Emotion ability/negativity might have been inflated. Therefore, it is necessary to verify the conclusions of this study with other measurement tools in the future.

Despite these limitations, our findings emphasize the importance of emotional dysregulation in the development of ODD symptoms in children. Therefore, improving children's emotion regulation has a positive effect on preventing the development of ODD in children and on avoiding the exacerbation of ODD symptoms.

## Conclusion

Our work provides promising preliminary evidence to explain the mechanisms linked with longitudinal changes in ODD symptoms in children from a self-regulation theory perspective. These findings support the current view that emotion regulation, particularly EL, is a core feature for the development of ODD symptoms. Additionally, we found differences in how teacher reported and parent reported ODD symptoms were related to children’s emotion regulation as a result of situational specificity, which underscores the need to collect information on children from multiple sources. Our study implies that educators and parents should appreciate children with ODD symptoms during school-age, as this is a critical period for emotion regulation development and the pathogenesis of ODD, which may further develop into a vicious cycle between emotion dysregulation and ODD if timely intervention is lacking.

## Data Availability

The datasets generated during and/or analyzed during the current study are available from the corresponding author on reasonable request.
